# The association of sleep disordered breathing with left ventricular remodeling in CAD patients: a cross-sectional study

**DOI:** 10.1186/s12872-017-0684-1

**Published:** 2017-09-18

**Authors:** Audrius Alonderis, Nijole Raskauskiene, Vaidute Gelziniene, Narseta Mickuviene, Julija Brozaitiene

**Affiliations:** Behavioral Medicine Institute, Lithuanian University of Health Sciences, Vyduno 4, 00135 Palanga, Lithuania

**Keywords:** Cad, Echocardiography, Hypertrophy, Left ventricular geometry, Polysomnography, Sleep apnea

## Abstract

**Background:**

There is still insufficient knowledge on the potential effect of mild to moderate sleep-disordered breathing (SDB) that is widely prevalent, often asymptomatic, and largely undiagnosed in patients with stable coronary artery disease (CAD). SDB affects 34% of men and 17% of women aged between 30 and 70. The objective of this study was to evaluate the association between SDB and left ventricular (LV) hypertrophy as well as structural remodeling in stable CAD patients.

**Methods:**

The study was based on a cross-sectional design. Echocardiography and polysomnography was performed in 772 patients with CAD and with untreated sleep apnea. All study participants underwent testing by Epworth Sleepiness Scale questionnaire. Their mean age, NYHA and left ventricular ejection fraction were, respectively: 57 ± 9 years, 2.1 ± 0.5 and 51 ± 8%, and 76% were men. Sleep apnea (SA) was defined as an apnea-hypopnea-index (AHI) ≥5 events/h, and, non-SA, as an AHI <5.

**Results:**

Sleep apnea was present in 39% of patients, and a large fraction of those patients had no complaints on excessive daytime sleepiness. The patients with SA were older, with higher body mass and higher prevalence of hypertension. LV hypertrophy (LVH), defined by allometrically corrected (LV mass/height^2.7^) gender-independent criteria, was more common among the patients with SA than those without (86% vs. 74%, *p* < 0.001). The frequency of LVH by wall thickness criteria (interventricular septal thickness or posterior wall thickness ≥ 12 mm: 49% vs. 33%, p < 0.001) and concentric LVH (61% vs. 47%, *p* = 0.001) was higher in CAD patients with SA. The patients with SA had significantly higher values of both interventricular septal thickness and posterior wall thickness. Multiple logistic regression analysis showed that even mild sleep apnea was an independent predictor for LVH by wall thickness criteria and concentric LVH (OR = 1.5; 95% CI 1.04–2.2 and OR = 1.9; 1.3–2.9 respectively).

**Conclusions:**

We concluded that unrecognized sleep apnea was highly prevalent among patients with stable CAD, and the majority of those patients did not report daytime sleepiness. Mild to moderate sleep apnea was associated with increased LV wall thickness, LV mass, and with higher prevalence of concentric LV hypertrophy independently of coexisting obesity, hypertension, diabetes mellitus or advancing age.

## Background

Coronary artery disease (CAD) is a major health issue in developed countries and constitutes a significant cause of death and disability. According to data from the Framingham Heart Study, a population-based longitudinal study, nearly one-half of males and one-third of females over 40 years of age will develop some manifestation of CAD [1]. Sleep-disordered breathing (SDB) and the related clinical syndrome, sleep apnea (SA), are highly prevalent in patients with ischemic heart disease and often remain undiagnosed, even after admission for myocardial infarction treatment [2, 3]. Sleep apnea is a condition in which breathing stops for more than ten seconds during sleep. Specific questions include whether SA is important in initiating the development of cardiac and vascular disease, whether SA in patients with established cardiovascular (CV) disease accelerates disease progression [4]. According to published data, atrial fibrillation, CAD, congestive heart failure, and arterial hypertension are clinical manifestations and are more common in patients with SA [2, 5–7]. SDB and nocturnal hypoxaemia are highly prevalent in stable heart failure with reduced left ventricular function and were identified as independent predictor of all-cause mortality in these patients [8]. Pathophysiology of acute coronary syndrome (ACS) deeply differs from stable angina, mainly due to peculiar features of plaque [9]. ACS, which is known to be characterized by a higher conventional CV risk, is caused by the loss of integrity of the protective covering of some atherosclerotic plaques, leading to thrombus formation and subsequent vessel obstruction [9, 10]. SA and sleep disruption have also been linked to an imbalance in circulatory thrombotic and anti-thrombotic activity [11]. Evidence linking SA and the severity of SDB to increased mortality and CV morbidity has been conflicting and inconclusive in both stable and unstable patients with established CAD [6, 12–17].

Abnormalities in left ventricular (LV) geometry, including LV hypertrophy (LVH), is both a target organ response to chronic arterial hypertension and other CV disorders; and LVH is an independent risk factor for coronary heart disease, heart failure, arrhythmias, including sudden cardiac death, stroke, and other major CV morbidity and mortality [16, 18–20]. Recurrent apneas during sleep lead to a sequence of events that, independently or in concert with other recognized risk factors, are likely to have harmful effects on CV structure and function. Several studies reported increased left-ventricular thickness or mass in association with SA but these relations were not significant after adjustment for body weight [21, 22].

Patients with SDB have a high prevalence of arterial hypertension and, more importantly, obesity, which represents the strongest predisposing factor for SDB [23–25]. Hypertrophy, defined as an increase in LV mass in relation to body size, is produced by an increase in chamber size, an increase in wall thickness, or both [26]. The relation between hypertension and obesity and its impact on LV geometry are known [27–33]. There is still insufficient knowledge on an impact of SA on LV geometry and on potential effect of mild to moderate sleep-disordered breathing, which is prevalent, often asymptomatic, and largely undiagnosed in stable CAD [12, 26].

The present study was performed to determine the association of SDB with LV geometry. We hypothesized, that in stable CAD patients even mild to moderate SA is an independent risk factor for: (1) a higher prevalence of LVH, by wall thickness criteria, with more pronounced both septal and posterior wall thickening; (2) a higher prevalence of concentric left ventricle than in patients without SA controlling for traditional recognized risk factors.

## Methods

### Design and setting

The study was a cross-sectional investigation of 1027 consecutive stable CAD patients attending the Clinic of Cardiovascular Rehabilitation, from October 2007 until December 2014 for routine cardiac rehabilitation program. Criterion for inclusion to the study are following: patients with CAD, men and women, from 18 years and older, consecutively referred to the rehabilitation clinic within two weeks after treatment for acute coronary syndromes (ACS), no current drug or mechanical treatment for SA. All patients were receiving standard treatment for secondary prevention of CAD and heart failure (HF) according to existing guidelines.

137 (13.3%) Of the patients were excluded from the study for meeting the criteria of being older than 80 years and having cognitive disorientation and communicative disabilities, having other severe diseases or has refused to participate in the program. In total, 887 (86.3%) Participants had successful echocardiographic data, 775 (75.4%) Participants had successful PSG of which 772 also had PSG data in conjunction with echocardiographic and ESS data (the other 115 with successful echocardiography data either elected not to undergo PSG or were ineligible due to medical reasons). Several echocardiograms and PSGs were technically inadequate (*n* = 6, 0.5%). Although the inclusion rate for eligible patients for study analysis was 772 (75%), the inclusion design was consecutive. Anthropometric and clinical profile of the patients not involved into the study did not differ significantly compared with the studied group. There were more cases of diabetes mellitus (23.5% Vs. 13.7%, *p* < 0.001) In studied patients than those in non-studied patients. The ratio of male patients was higher in not studied group (81.2% Vs. 73.6%, *p* = 0.001).

### Ethic

The study and its consent procedures were approved by the Lithuanian Bioethics Committee (Certificate No. BE-2-21 issued at 2007–04-13) and conform to the ethical guidelines of the 2000 Declaration of Helsinki. Written informed consent was obtained from each study patient.

### Patient assessment

All patients were evaluated for demographic characteristics (age, gender), CAD risk factors - diabetes mellitus, smoking, arterial hypertension, body mass index (BMI), and clinical characteristics, including New York Heart Association (NYHA) functional class, diabetes mellitus and use of medications. Hypertension was defined as a blood pressure > 140/90 mmHg measuring BP using sphygmomanometer or if the subject was receiving antihypertensive medications. All patients underwent overnight-polysomnography and were classified as sleep apneics or controls according to the apnea-hypopnea index.

### Anthropometry

Weight and height for study subjects was measured in indoor clothing and without shoes. BMI was calculated as weight (in kg) divided by height (in m^2^). Measured weight and height were used to calculate BMI, and categorized as follows: < 25, 25–29.9, and ≥30 kg/m^2^, to represent normal, overweight, and obese (class I (30–34.9); class II (35–39.9), class III ≥40 kg/m^2^) individuals for all analyses.

### Polysomnography, classification of SA

Overnight fully attended polysomnography (PSG) monitoring was performed with the “Alice 4” Polysomnography System (Respironics Inc., USA) in the Sleep laboratory using standard recording techniques according to AASM (American Academy of Sleep Medicine) and precise protocol of PSG monitoring [34]. Surface electrodes were applied to perform electroencephalogram, chin electromyogram, electrocardiogram, and electrooculography. Airflow was monitored using a thermistor placed at the nose and mouth, and arterial oxygen saturation (SaO_2_) was recorded continuously with a pulse oximeter. Apnea was defined as the disappearance of airflow for over 10 s; hypopnea was defined as a 50% or greater decrease in airflow lasting for more than 10 s associated with arousal or a 3% or greater decrease in arterial oxygen saturation from the baseline level. Physiological variables collected from the polysomnograms included the number of central apneas, obstructive or mixed apneas, mean oxygen saturation during sleep; and the max oxygen desaturation during sleep, oxygen desaturation index (ODI), respiration disturbance index (in total sleep time, AHI). The most common type of SA is obstructive sleep apnea (OSA), and much of the pathophysiologic understanding of SA relies on studies of OSA [4]. In our study the influence of central and obstructive events was not separately analysed.

The apnea-hypopnea index (AHI) was calculated as the total number of apnea and hypopnea episodes per hour of sleep [34]. According to the American Academy of Sleep Medicine criteria [34] SA was defined as five or more episodes of apnea and hypopnea per hour of sleep (AHI ≥ 5 n/h) and classified as mild (AHI 5–15), moderate (AHI 16–30), or severe (AHI > 30 n/h). Patients with AHI < 5 were classified as not having SA (non-SA group).

*Epworth Sleepiness Scale (ESS)* - was used for evaluation of the subjective excessive daytime sleepiness (EDS). Briefly, the patient rates the probability of dozing (0 to 3) in eight different situations. No specific time frame is specified. The ESS score represents the sum of individual items, and ranges from 0 to 24. A score 10 and more is considered as positive for the presence of excessive daytime sleepiness [35].

### Echocardiography recording and analysis

Echocardiographic evaluation of the patients was blinded to the results of the PSG. Echocardiography studies were performed using Esaote echocardiography System (My Lab 50 Xvision, Italy). LV systolic function was assessed by LV ejection fraction (LVEF) using the modified biplane Simpson’s method with apical two- and four-chamber views. It was defined as reduced LVEF while LVEF less than 45%. LV end-diastolic internal dimension (LVEDD), LV end-systolic internal dimension (LVESD), interventricular septal thickness (IVST), and LV posterior wall thickness (LVPWT) were determined from M-mode measurements according to the American Society of Echocardiography (ASE) guidelines [36]. The LV mass was calculated using the ASE convention [37, 38]:

$$ \mathrm{LVM}=0.8\left[1.04\left\{{\left(\mathrm{IVST}+\mathrm{LVPWT}+\mathrm{LVEDD}\right)}^3\hbox{--} {\mathrm{LVEDD}}^3\right\}\right]+0.6 $$

LVM was divided by height^2.7^ and by body surface area (BSA) to obtain left ventricular mass index (LVMI). Because LVM was most strongly correlated with BMI and there is not a specific value adjusted for obesity, for this reason we adopted the most commonly used: LVM adjusted according to height^2.7^ (LVM/height^2.7^) [32] which is proposed to be similarly sensitive in detecting obesity-related and -unrelated LVH. The height^2.7^ index was also important for the subcategorization of patients according to the presence of concentric or eccentric LVH because the prognostic value of such subcategorization was apparent only when the height^2.7^-based criterion was applied [39].

LVH was defined as gender-neutral cutoff value for LVM indexed for height > 51 g/m^2.7^ [40, 41]. Relative wall thickness (RWT) was computed by the ratio of sum of IVST and LVPWT to LVEDD. LV structure was categorized as normal (RWT ≤0.42 and no LVH), concentric remodeling (RWT >0.42 and no LVH), concentric hypertrophy (RWT >0.42 and LVH) and eccentric hypertrophy (RWT ≤0.42 and LVH) [36]. In addition, for the purposes of this study, we defined the prevalence of LVH on the basis of wall thickness criteria (IVST or LVPWT ≥12 mm) [22, 37].

### Statistical analysis

Quantitative variables are expressed as means ± SD or medians (interquartile ranges [IQRs]) or in percentages, when appropriate. The Kolmogorov-Smirnov test was used to assess the normal distribution of continuous variables. To compare basic characteristics between non-SA and SA (AHI ≥ 5) groups the significance of differences was assessed using Mann–Whitney tests (or t tests, if data were normally distributed) or chi-squared tests, respectively. Analysis of variance (ANOVA) was used to determine whether there are any statistically significant differences between the means of three (non-SA group excluded) or four independent SA severity (coded as 1 = non-SA, 2 = mild, 3 = moderate, 4 = severe) groups. For the linear trend the *p* value for F-ratio was presented. A Bonferroni correction was applied to post hoc pairwise comparisons of means.

To further substantiate the notion that mild-severe SA was an independent determinant of LVH by wall thickness criteria or concentric LV geometry (dependent variable coded 0 = absent/1 = present), bivariate analysis and multiple logistic regression analyses were performed. In the multiple logistic regression analysis, statistically significant variables in the bivariate analysis, as well as the non-significant variables with suggested clinical relevance, were included. The candidate explanatory (independent) variables for entering in the multiple regression model were hypoxemia during sleep SaO_2_ and age, AHI (4 categories), BMI (4 categories), age over 60, sex, hypertension, diabetes mellitus, NYHA class, current smoking, history of MI as well as LVEF lower than 45%, excessive daytime sleepiness (ESS ≥ 10). Because daytime sleepiness is common in patients with SA, this factor was taken into account as potential confounding factor. The goodness of fit and Nagelkerke R^2^ for these models presented. Forward stepwise selection was then performed to identify factors independently associated with LVH. Data were analyzed with SPSS 17.0 statistical software (SPSS Inc., Chicago, Illinois, USA). A *p*-value <0.05 was considered significant.

## Results

### Clinical characteristics

Patients were predominantly male (76%), mean age was 57 ± 9 years (ranged from 32 to 81 years). The median value of the AHI in the sample was 3.4 and the interquartile range (25th percentile to 75th percentile) was 1 to 9. Sleep apnea defined as five or more episodes of apnea and hypopnea per hour of sleep (AHI ≥ 5 n/h) was present in 300 (39%) of patients and 175 (22.7%) of patients had LVEF ≤ 45%.

### General characteristics comparison according to SA status

Compared to non-SA patients, individuals with SA were on average older (SA: 59 ± 9 years vs. non-SA: 56 ± 9 years, *p* < 0.001), more frequently male (83% vs. 72%; *p* = 0.004), and had a higher prevalence of traditional CV risk factors such as obesity and hypertension (53% vs. 43% and 91% vs. 78%, respectively; both *p* < 0.01). Current smoking was more common in the non-SA patients than that in patients with SA. Almost equal proportions were in NYHA classes (χ^2^ = 4.8 *p* = 0.09) (Table 1). We also estimated that 42% of men and 28% of women had an AHI ≥5 (χ^2^ = 12.3, *p* < 0.001).

**Table 1:** Univariate analysis of demographic, clinical characteristics and comorbidities of the CAD patients according to SA status

Characteristic	Total sample *N* = 772	Non-SA AHI: <5 *n* = 472	SA AHI: ≥5 *n* = 300	p
Age, years	57 ± 9	56 ± 9	59 ± 9	<0.001
Age over 60 years, n (%)	344 (47)	191 (40)	153 (50)	χ^2^ = 8.2 p = 0.004
Male gender, n (%)	586 (76)	338 (72)	248 (83)	0.001
BMI, kg/m^2^	30.0 ± 4.8	29.4 ± 4.6	31.0 ± 5.0	<0.001
BMI, n (%):				χ^2^ = 11.8 p = 0.003
Normal (<25 kg/m^2^)	104 (13.5)	78 (16.5)	26 (8.7)	
Overweight (25–24.9 kg/m^2^)	305 (39.5)	189 (40.0)	116 (38.7)	
Obese (≥30 kg/m^2^)	363 (47.0)	205 (43.4)	158 (52.7)	
Coronary artery disease (CAD), n (%):				χ^2^ = 3.8 *p* = 0.43
Stable angina pectoris	230 (29.8)	140 (29.7)	90 (30.0)	
First myocardial infarction	729 (94.4)	278 (58.8)	173 (57.7)	
Previous myocardial infarction	91 (11.7)	54 (11.5)	37 (12.3)	
NYHA class, n (%):				χ^2^ = 4.8 p = 0.09
I	65 (8.4)	48 (10.2)	17 (5.7)	
II	598 (77.5)	359 (76.4)	239 (79.9)	
III	106 (13.7)	63 (13.4)	43 (14.4)	
LVEF ≤45%, n (%)	175 (22.7)	95 (20.1)	80 (26.7)	χ^2^ = 4.5 p = 0.034
Hypertension n (%)	641 (83.0)	369 (78.2)	272 (90.7)	χ^2^ = 20 p < 0.001
Diabetes mellitus, n (%)	165 (21.4)	102 (21.6)	63 (21.0)	χ^2^ = 0.04 *p* = 0.84
History of smoking, n (%)	97 (12.6)	67 (14.3)	30 (10.0)	χ^2^ = 2.9 *p* = 0.08
Median (IQR) Epworth Sleepiness Scale, score	6 (4–9)	6 (3–8)	6 (4–9)	0.006^a^
Excessive day time sleepiness ESS > 10, n (%)	131 (17)	74 (15.7)	57 (19.0)	χ^2^ = 1.4 p = 0.14

Values are mean ± standard deviation, or number of patients (%) unless stated otherwise

*BMI* body mass index, *ESS* Epworth Sleepiness Scale, *LVEF* left ventricular ejection fraction, *IRQ* interquartile range, *SA* sleep apnea, *AHI* apnea-hypopnea index, *NYHA* New York Heart Association

^a^Mann-Whitney U test

ESS ranged from 0 to 18 with a median of 6 (interquartile range 4–9). The patients with SA had higher scores on the ESS than those non-SA patients (*p* = 0.006). No statistically significant between-group differences were found for frequency of excessive daytime sleepiness (ESS ≥ 10) (respectively 16% and 19%; *p* = 0.14) (Table 1). The mean ESS score was within the normal range in both groups (6.0 ± 3.5 vs. 6.7 ± 3.5). ACE inhibitors/angiotensin II receptor blockers and beta-blockers were prescribed to 81% and 89% of study patients, respectively. Antihypertensive and antiagregant drugs were used by larger number of patients with SA than non-SA patients (*p* = 0.012 and *p* = 0.003, respectively; data not shown).

### Clinical, polysomnographic and echocardiographic characteristics according to SA severity

There were no statistically significant differences in their mean age and sex in SA patients groups according to severity of SA. Prevalence of NYHA class, percentage of hypertension and LVEF ≤ 45%, as well as diabetes mellitus, history of MI, smoking history did not differ between mild, moderate and severe SA patients groups. However, BMI was higher in the severe SA patients than that in patients with moderate (*p* = 0.025) and mild (*p* = 0.002) SA (Table 2).

**Table 2:** Basic characteristics of the CAD patients with SA according to SA severity

	SA severity group			
Variable	Mild AHI: 5–14.9 n = 192	Moderate AHI: 15–29.9 *n* = 59	Severe AHI: ≥30 *n* = 49	p for trend^a^
	1	2	3	
Age, years	59.1 ± 8.7	59.7 ± 9.3	60.9 ± 7.3	0.401
Age over 60 years, n (%)	88 (47)	32 (54)	33 (67)	0.037
Male, n (%)	148 (79.1)	52 (88.1)	44 (89.8)	0.087
BMI, kg/m^2^	30.5 ± 4.9	30.7 ± 4.5	33.2 ± 5.5^b^	0.003
Hypertension, n (%)	171 (91.4)	53 (89.8)	45 (91.8)	0.965
Diabetes mellitus, n (%)	38 (20.3)	9 (15.3)	15 (30.6)	0.139
NYHA class, mean	2.05 ± 0.43	2.14 ± 0.47	2.16 ± 0.47	0.203
Myocardial infarction, n (%)	133 (71)	39 (66)	16.9 (71)	0.746
LVEF ≤ 45%, n (%)	53 (28.3)	13 (22.0)	13 (26.5)	0.600
Smoking history, n (%)	19 (10.2)	7 (11.9)	3 (6.1)	0.588

Values are mean ± standard deviation, or number of patients (%)

*SA* sleep apnea, *AHI* apnea-hypopnea index, *BMI* body mass index, *LVEF* left ventricular ejection fraction, *NYHA* New York Heart Association

^a^ANOVA or χ^2^ (differences between different SA severity groups)

^b^p < 0.002 severe SA vs. mild SA and p = 0.025 severe SA vs. moderate SA

There was (as expected) a gradual increase in mean AHI and ODI with increase in SA severity (p for trend < 0.001). Patients with severe SA had both the lowest average SaO_2_ and highest maximal oxygen desaturation (*p* < 0.001). Severity of SA did not manifest by sleepiness (p for trend = 0.062). Even in the most severe apnea category (AHI ≥ 30) the mean ESS score statistically significant differ from that in non-SA patients (being within the normal range 7.6 ± 3.8) (Table 3).

**Table 3:** Polysomnography characteristics of the CAD patients according to SA severity

		SA severity group			
Variable	non-SA AHI < 5 n = 472	Mild AHI: 5–14.9 *n* = 192	Moderate AHI: 15–29.9 n = 59	Severe AHI: ≥30 n = 49	p for trend^c^
		1	2	3	
AHI, n/h	1.7 ± 1.5^a^	9.0 ± 2.9	21.7 ± 4.5	44.3 ± 10.8	<0.001
ODI, %	2.6 ± 7.7^a^	8.1 ± 12.4^23^	14.0 ± 17.4^13^	35.1 ± 25.1^12^	<0.001
Average SaO_2_, %	94.5 ± 1.7^a^	93.9 ± 1.8^3^	93.4 ± 2.8^3^	91.9 ± 4.9^12^	0.001
DeSaO_2_ max, %	6.7 ± 8.3^a^	12.0 ± 8.0^3^.	15.2 ± 10.5^3^	24.6 ± 17.5^12^	<0.001
ESS score	6.0 ± 3.5^b^	6.7 ± 3.5	6.0 ± 3.2	7.6 ± 3.8	0.062
ESS > 10, n (%)	74 (15.7)	34 (17.7)	11 (18.6)	12 (24.5)	0.556

Data are presented as mean ± SD or number of patients (%)

*SA* sleep apnea, *AHI* apnea-hypopnea index, events per hour, *ODI* oxygen desaturation index, *SaO*_*2*_ saturation of nocturnal arterial oxygen, *DeSaO*_*2*_ *max* maximal oxygen desaturation, *ESS* Epworth sleepiness scale

^a^p < 0.01 between non-SA and every SA groups

^b^ *p* < 0.05 between non-SA and Sever SA (ANOVA for all four groups, post hoc with Bonferroni correction)

^c^ANOVA or χ^2^ test for SA severity groups, non-SA group was excluded from this analysis

^123^the number in superscript indicates the SA severity groups, with significant differences p < 0.05 (ANOVA for three SA severity groups, post hoc with Bonferroni correction)

We found no statistically significant differences in a frequency of EDS among patients without (15.7%) and with SA despite the severity (17.7% to 24.5%, *p* = 0.556) (Table 3).

Reduced LVEF (≤45%) was more frequent in the patients with SA than those in non-SA patients (26.7% and 20.1% respectively, *p* = 0.034) (Table 1). There were no significant differences in LVEF (mean ejection fraction) of patients included in any of the groups of SA severity (p _for trend_ = 0.38), suggesting that SA severity was not affecting changes in their LV systolic function. Post hoc analysis (data not shown) demonstrated that only the LVEDD was marginally significantly higher in severe SA patients than those in mild SA patients (mild-SA vs. severe-SA *p* = 0.044 (not shown), model F = 3.03 p _for trend_ = 0.05) (Table 4).

**Table 4:** Basic echocardiographic measurements of the left ventricle in SA and non-SA patients

Variable	Non-SA AHI: <5 *n* = 472	SA AHI: ≥5 n = 300	t test, Mann–Whitney or χ^2^ test	p for trend^a^
Thickness and diameters				
Left atrium, cm	3.8 ± 0.5	4.1 ± 2.2	<0.001	0.110
LVPWT, mm	11.1 ± 1.1	11.6 ± 1.1	<0.001	0.904
IVST, mm	11.2 ± 1.6	11.8 ± 1.7	<0.001	0.829
LVEDD, mm	50.3 ± 5.1	51.6 ± 5.6	0.003	0050
LVESD, mm	33.8 ± 6.1	34.7 ± 5.7	0.013	0.052
LVWT, mm	22.3 ± 2.4	23.4 ± 2.4	<0.001	0.829
RWT	0.45 ± 0.06	0.45 ± 0.06	0.149	0.232
LVH by wall thickness criteria, n (%)	151 (33.4)	138 (49.3)	<0.001	0.268
IVST ≥ 12 mm	126 (27.9)	118 (42.1)	<0.001	0.381
LVPWT ≥ 12 mm	79 (17.5)	89 (31.7)	<0.001	0.593
LV mass index ≥51 g/m^2.7^, n (%)	333 (74.0)	241 (86.4)	<0.001	0.348
RWT > 42, n (%)	285 (63.3)	195 (69.9)	0.077	0.348
Concentric LVH, n (%)	214 (47.1)	168 (60.9)	0.001	0.438
Left ventricular mass				
LVM g	256.4 ± 63.4	285.9 ± 68.5	<0.001	0.210
LVMI g/m^2^	128.7 ± 26.9	139.5 ± 29.4	<0.001	0.765
LVMI g/m^2.7^	59.7 ± 13.9	66.1 ± 15.4	<0.001	0.550
LVEF, %	52.1 ± 8.4	50.4 ± 8.7	0.007	0.380
LVEF ≤ 45%, n (%)	95 (20.1)	80 (26.7)	0.034	0.135

Data are presented as mean ± SD or number of patients (%)

*SD* indicates standard deviation, *SA* sleep apnea, *AHI* apnea-hypopnea index, *IVST* interventricular septum thickness in diastole, *LVPWT* left ventricular posterior wall thickness in diastole, *LVEDD* left ventricular end-diastolic diameter, *LVESD* left ventricular end-systolic diameter, *LVWT* left ventricular wall thickness, *RWT* relative wall thickness, *LVH* left ventricular hypertrophy, *LVM* left ventricular mass, *LVMI* left ventricular mass index, *LVEF* LV ejection fraction

^a^ANOVA or χ^2^ for SA severity groups, data not shown, non- SA patients were excluded from this analysis

There were not found statistically significant differences in left atrium diameter, in thickness of IVST and LVPWT, in value of LVM and LVMI (g/m^−2^ and g/m^2.7^) of patients included in any of the groups of SA severity. This suggests that SA severity according to AHI categories was not inducing changes in their LV thickness and mass (ANOVA for SA severity groups all p _for trend_ > 0.05, Table 4).

### Left ventricular hypertrophy

**Fig. 1:**
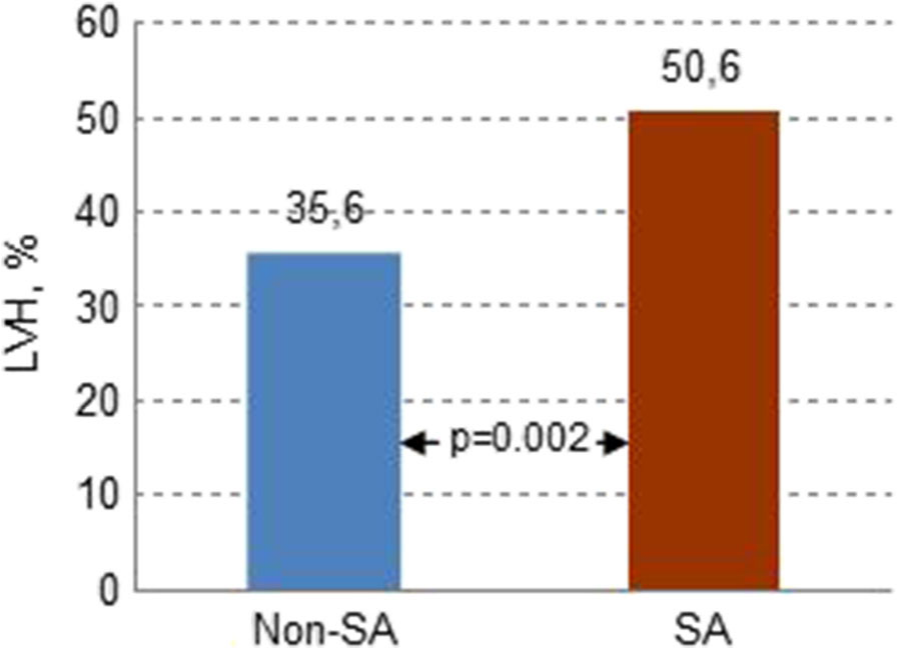
Prevalence of left ventricular hypertrophy on the basis of wall thickness criteria (interventricular septal thickness or posteriorwall thickness ≥ 12 mm). LVH = left ventricular hypertrophy; SA = sleep apnea

In univariate analysis the CAD patients with SA had a 1.9-fold higher prevalence of LVH on the basis of wall thickness criteria (IVST or LVPWT ≥12 mm) compared to non-SA patients (49.3 vs. 33.4%; odds ratio, OR = 1.9; 95% confidence interval (CI), 1.4–2.6; *p* < 0.001) (Table 4, Fig. 1).

Left atrium diameter, IVST and LVPWT was significantly higher in patients with SA, than those in non-SA patients (all p < 0.001). However, no between-group differences were found for RWT (Table 4).

LVH defined by allometrically corrected (LV mass/height^2.7^) gender-independent criteria was present in 86% of the SA patients compared to 74% in non-SA patients group (*p* < 0.001).

### Distribution of LV geometrical pattern

Some form of LV geometric remodeling was present in 92% of patients, with concentric LVH as the most common geometric abnormality. Eccentric LVH occurred in 26.3% (*n* = 192), concentric LVH in 52.4% (*n* = 382) of patients, concentric LV remodeling in 13.4% (*n* = 98). Eccentric LVH is much less prevalent than concentric hypertrophy.

**Fig. 2:**
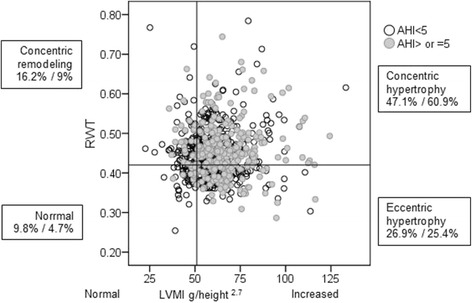
Frequency distribution of the four different LV–geometrical patterns in patients with vs without sleep apnea. The spreadsheet showed the distribution of LV mass index versus relative wall thickness (RWT). Apnea-hypopnea index (AHI) ≥5 n/h = sleep apnea. Left ventricular geometry, including concentric remodeling as well as eccentric and concentric hypertrophy based on normal/high RWT and left ventricular hypertrophy (LVH) based on left ventricular mass index (LVMI g/height ^2.7^) criteria. The reference lines indicate the gender-independent LVH cut-off, (51 g/m ^2.7^, vertical line), and the upper limit of RWT (0.42, horizontal line). In boxes: geometric pattern, percentage: without sleep apnea/with sleep apnea; χ^2^-test df = 3 *p* < 0.001

The frequency distribution of the LV geometric groups indicated significant differences between SA and non-SA patients (χ^2^ = 18.9; p < 0.001) (Fig. 2). Percentage of concentric hypertrophy defined as the combination of an increased RWT and an increased LVMI (LVM/height^2.7^) was 60.9% in CAD patients with SA compared to 47.1% (*p* < 0.001) in non-SA patients (Fig. 2).

LVH (high LVMI) is traditionally two right panels designate subjects with increased LVMI (Fig. 2). As it described in Table 4 pattern of concentric remodeling – comprising high RWT without an absolute increase in mass index, pattern of concentric hypertrophy depend on increased LV wall thickness and chamber dilation is present.

### Determinants of LV hypertrophy in the CAD patients

#### Predictors of LVH on the basis of wall thickness criteria

**Fig. 3:**
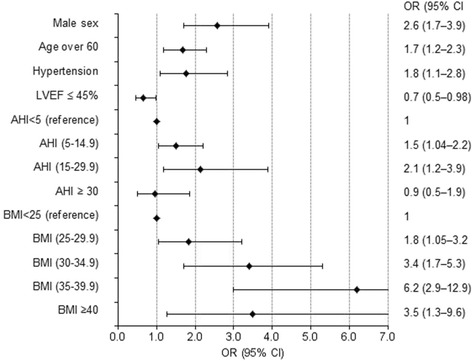
Cross sectional predictors of left ventricular hypertrophy on the basis of wall thickness criteria (interventricular septal thickness or posterior wall thickness ≥ 12 mm). The independent variables entered in the multiple regression model were: age, sex, ESS score, hypoxemia during sleep SaO_2_, age over 60, AHI (4 categories), BMI (4 categories), NYHA class, hypertension, diabetes mellitus, current smoking, history of MI as well as LVEF lower than 45% and excessive daytime sleepiness (ESS ≥ 10). Model: Hosmer & Lemeshow test χ^2^ = 8.9 df = 8 *p* = 0.349; Nagelkerke R^2^ = 0.213. Abbreviations: ESS = Epworth sleepiness scale, LVEF = left ventricular ejection fraction; AHI = apnea-hypopnea index; BMI = body mass index; OR = odds ratio; CI = confidence interval

Multiple logistic regression analysis highlighted cross sectional predictors of LVH on the basis of wall thickness criteria (interventricular septal thickness or posterior wall thickness ≥ 12 mm) such as male sex (OR = 2.6) *p* < 0.001), hypertension (OR = 1.8 *p* = 0.016), age over 60 years (OR = 1.7 *p* = 0.002), LVEF ≤45% (OR = 0.7 p < 0.001) and AHI (for trend *p* = 0.029): mild SA versus non-SA (OR = 1.5; *p* = 0.030), moderate SA versus non-SA (OR = 2.1; *p* = 0.012) (Fig. 3).

The prevalence of LVH on the basis of wall thickness criteria was lower in patients with LVEF ≤ 45% and did not differ significantly between the patients with severe SA (AHI ≥ 30) and non-SA (OR = 0.96; *p* = 0.91). Furthermore, the risk progressively increased with higher BMI values (p for trend <0.001) but this increase slowed down in patients with BMI ≥ 40 versus normal (OR = 3.5 95% CI, 1.3–9.6; p < 0.001) (Fig. 3).

#### Predictors of concentric LVH

Mild SA (AHI = 5–14.9 /h), performing multiple logistic regression analysis, was found as a strong independent cross sectional predictor *of concentric LVH* in the CAD patients (OR = 1.9). The risk of concentric hypertrophy did not increase with the greater level of SA severity.

**Fig. 4:**
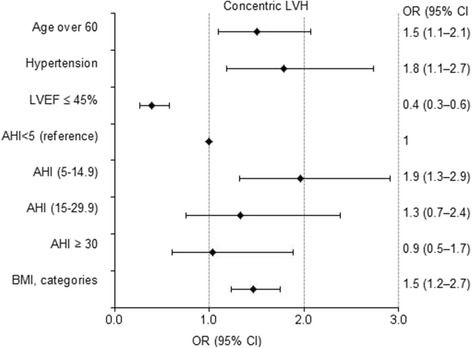
Cross sectional predictors of concentric left ventricular hypertrophy. The independent variables entered in the multiple regression model were: age, sex, ESS score, hypoxemia during sleep SaO_2_, age over 60, AHI (4 categories), BMI (ORs per 5 kg/m^2^ higher BMI above 25 kg/m^2^), NYHA class, hypertension, diabetes mellitus, current smoking, history of MI as well as LVEF lower than 45% and excessive daytime sleepiness (ESS ≥ 10). Model: Hosmer & Lemeshow test χ^2^ = 5.1 df = 8 *p* = 0.746; Nagelkerke R^2^ = 0.132. Abbreviations: ESS = Epworth sleepiness scale; LVEF; left ventricular ejection fraction; AHI = apnea-hypopnea index; BMI = body mass index; OR = odds ratio; CI = confidence interval

For BMI increase every 5 kg/m^2^ (e.g., moving from normal weight to overweight, or from overweight to mild obesity), the risk of concentric LVH also increased by 50% (OR = 1.5). Such factors as gender, history of smoking, diabetes mellitus were not statistically significant (Fig. 4).

## Discussion

### Summary of findings

Previous studies, that often lacked adjustment for important cardiovascular risk factors suggested that SDB was associated with changes in heart structure, including LV hypertrophy. The present study was performed in a large sample of consecutive *stable CAD patients* characterized by a relatively high degree of comorbidity. This study extends these results with the novel finding that *even mild level* of SDB is strongly associated with LVH, with increase LV wall thickness (both interventricular septum and posterior wall) and concentric LVH, independently of coexisting traditionally recognized risk factors. The risk of concentric hypertrophy did not increase with greater levels of SDB severity.

These results could suggest that the effect of SA on LVH risk is fully realized at certain SA severity level. As hypothesized, there was almost 2-fold increase in risk of having LVH on the basis of wall thickness criteria, and 1.5-fold higher risk of concentric hypertrophy, adjusted for the traditionally recognized risk indicators in CAD patients with the disordered breathing.

Although patients with SA had a higher mean value of the ESS than non-SA patients, this value was lower than the reference value for excessive sleepiness (ESS ≥10). Therefore, this value did not seem to be a determining factor in the diagnosis of SA in these patients. The limitations of ESS are known in cardiovascular patients and the existence of patients with severe SA without excessive sleepiness is controversial [41, 42].

Though we do not claim any causal relation our results could suggest that even mild SA had additive effect in changes to the heart shape, similar to the effects of both hypertension and obesity. This effect includes increased mass and thickening of the heart wall. As patients with stable CAD and with SA often do not show typical SA symptoms, the presence of one or more established predictors of LVH *on the basis of wall thickness criteria or concentric LVH,* such as male sex, older age, higher BMI, ejection fraction >45%, or SA may *constitute independent risk factors* for adverse long-term outcomes. The increase recognition of SA as an independent, additive or even synergistic risk factor for CAD brings a growing need for early identification of high-risk individuals and a consensus regarding well defined treatment strategies in such patients.

### The prevalence of SA in CAD patients

The data in our cross-sectional study indicates that undiagnosed SA was highly prevalent (39%) among patients with CAD. The prevalence of SA in patients with CAD was higher than in normal population, as stated by numerous studies, and was in range or higher than in our study. The studies reported a wide range of prevalence, ranging from 26% to 66%, partially explained by the different value of AHI used to establish the diagnosis of SA in the different studies [3, 5, 41].

There is some evidence suggesting that the prevalence and the severity of SA are modified along the CAD evolution. Mooe et al. reported that clinically significant sleep apnea (AHI >10) was documented in 37% of investigated 142 patients with stable angina pectoris and angiographically confirmed CAD [43]. Peker et al. reported 30% prevalence of SA in patients admitted with an acute coronary syndrome, while Areias et al. claimed SA to be present in 43% of the study patients [12, 17, 41]. In CAD patients immediately after an acute myocardial infarction and after clinical stabilization of unstable angina pectoris Moruzzi et al. observed a significantly higher AHI compared to stable angina pectoris patients [12, 44]. In the study conducted in Greece, the authors concluded that there was a high prevalence of SA during the acute phase of coronary syndrome ACS (54%); this prevalence decreased 6 months after ACS and persisted in 21% of the patients, which indicates that this abnormality may be transient [41, 45]. Recently, Fox et al. (2016) investigating the prevalence of SDB in cardiac rehabilitation patients, reported a prevalence of SDB (AHI ≥ 15) by different cardiac condition. They found that patients who had undergone coronary artery bypass graft (CABG) had a higher prevalence of SDB and more severe SDB compared to patients with stable CAD or ACS (prevalence of 53%, 46% and 45% respectively) [46]. As noted, patients with heart failure and SA (in particular CSA) do not generally present with symptoms that distinguish them from heart failure patients without SA. Arzt et al. (2016) reported a prevalence of 46% in a large representative population of patients with stable chronic HF receiving optimized medical treatment [47].

In general, there is a stronger relationship between SDB and CAD in clinical cohorts than in the general population because clinical cohort studies are particularly influenced by comorbidity and confounding factors, including obesity, hypertension, smoking, and hyperlipidemia. This circumstance may also suggest that SDB constitutes an additive or synergistic risk factor for development of CAD [14, 16].

### Cardiac remodeling in hypertension and obesity

Despite the high prevalence of SA reported in patients with CAD, it is frequently underdiagnosed [12]. In addition, comorbidities such as obesity and hypertension that coexist with the majority of SA patients make it more difficult to assess the independent risk of SA on vascular disease. *Both hypertension and obesity* strongly affect cardiovascular structure and function. Since obesity and hypertension are closely linked and often coexist, while obesity is an independent risk factor for arterial hypertension these two disorders often exert a dual burden on the left ventricle, leading to both dilatation and hypertrophy [30] as well as markedly increasing the premature risk of CV disease [19]. The presence of hypertension in patients with SA likely contributes to the association with LVH or increased wall thickness [48]. Data supporting a possible cause and effect relationship between SA and LVH were published by Cloward et al., who found that 6 months of nocturnal positive airway pressure administration to patients with severe OSA was associated with a significant reduction in LV wall thickness [49].

Many studies have shown that obesity is associated with LVH, a potential contributor to heart failure [29]. It is unclear to what extent LVH results directly from obesity or from associated conditions, such as hypertension, impaired glucose homeostasis, or obstructive sleep apnea [23]. Early studies from Ochsner demonstrated that the cardiac adaptation to these two disorders differed considerably [19]. Hypertension causes fundamental response to an isolated increase in pressure load or afterload such as an increase in LV wall thickening without chamber dilatation. On the other hand, obesity leads to LV chamber dilatation with only minimal increases in LV wall thickness. Cardiac remodeling in obesity is characterized by concentric geometry at least as often as eccentric geometry [33]. However, some investigators have found evidence of eccentric LVH (an increase in cavity volume that is greater than the increase in wall thickness) in obese subjects [30, 31, 48].

In fact, LVH in SDB is as closely related to the severity of the sleep disturbances as it is to the levels of blood pressure and BMI [23]. Our study findings also confirm the strong independent association between concentric LVH and obesity at any value of BMI. It helps to dispel the notion that LVH in this condition is exclusively eccentric. When obesity and hypertension coexist, there is a substantial increase in the prevalence of LVH at any value of BMI confirmed in many studies [23, 33, 49]. However, the increase in LV mass/height^2.7^ is more pronounced in patients with hypertension than in those without [33]. MacMahon and colleagues demonstrated that weight loss in hypertensive subjects with overweight reduced LVM and posterior wall thickness [50]. In contrast, Niroumand et al. concluded that SA is not associated with increased LVM independently from obesity, hypertension or advancing age [21].

### LV geometry

We found that male sex, age 60+, hypertension, higher BMI, higher than 45% LVEF and SA were independent predictors of LVH *on the basis of wall thickness criteria and predictors of concentric LVH (w*ith the exception of sex). These indicators showed additive effects of SA and are consistent with those of other studies. Drager et al. (2007) looked at arterial stiffness by measuring pulse wave velocity and heart structure in OSA patients with and without hypertension. They demonstrated that patients with untreated OSA had increased left atrial diameter, septal wall thickness, LV posterior wall thickness, LV mass index, and LVH, even controlling for cardiovascular risk factors [51]. These indicators showed additive effects of hypertension and OSA.

SDB produces a series of mechanical, hemodynamic, chemical, neural, and inflammatory responses (i.e. intermittent apnea-induced hypoxia, hypercapnia, increases in sympathetic drive and LV myocardial afterload, daytime hypertension, loss of vagal heart rate regulation and systemic vasculature remodeling) [23, 26, 49]. These strongly impact the CV system, being all potent stimuli to myocyte necrosis and apoptosis, myocardial ischemia, and finally, predict development of concentric LV geometry [26].

The associations between SDB and LV geometry have been previously investigated in a lot of studies conducted on patients with obesity and hypertension, yet the results are irreconcilable [21, 23, 26, 29, 31]. Both overweight and increased blood pressure determine a state of chronic myocardial LV overload and represent two of the main predisposing factors for LVH and systolic dysfunction. In theory, the two conditions are hemodynamically different and should lead to different LV geometry adaptations [30]. In clinical practice, such dichotomy is less evident because obesity and hypertension lead to heterogenic and unpredictable changes in LV geometry [18, 19, 23].

Although less well established than for hypertension, there is growing evidence that SA is a risk factor for incident diabetes [52]. In addition, some reports have demonstrated metabolic syndrome independent association of SA with LV structure [53, 54]. The relative absence of effects of diabetes mellitus on LVM in our study could be explained by relatively low prevalence of this CAD risk factor in our patient population, and the fact that this prevalence was similar in patients with SA and non-SA (21%). The impact of diabetes mellitus on LVM is predominantly through an interaction with obesity and age rather than a direct and independent effect. After adjusting for confounding factors there was no difference between diabetic and non-diabetic participants with regard to apnea-hypopnea events [14, 52].

Despite significant scientific evidence pointing to SA as an emerging CV risk factor, SA is still underdiagnosed in several cardiology subspecialties (hypertension, coronary, arrhythmia, heart failure, valvular heart disease) and consequently undertreated [55]. On account of the high prevalence of SA in the general population, the sleep medicine field is confronted by a demanding challenge to provide sufficient resources for the management of this disorder. In spite of the differences in the diagnostic procedures as well as different AHI cut-off values for definitions of SA, there seems to be enough evidence to warrant overnight sleep screening in individuals with CAD, given that concomitant SA may worsen long-term outcomes. Furthermore, several subsequent studies have demonstrated that patients with untreated severe SA exhibit an increased incidence of fatal and nonfatal cardiovascular events (including MI and ACS) compared with those without SA or even with those with untreated mild to moderate SA [6, 14, 16]. In a longitudinal study of 408 patients with CAD, Mooe and co-workers (2001) noted that SA independently increased the risk for incident cerebrovascular events [13]. Yet such an association suggests but does not demonstrate a causal relationship, and the role of treating SA to prevent CV diseases remains unclear [6]. SA is prevalent in our study population, especially *in its mild form*. Although severe SA is associated with adverse health consequences, the association with mild disease is unclear. Reports differ regarding the clinical relevance of mild SA. Improved diagnostic techniques and evidence based approaches to management in mild SA require further research. Exploring potential causes and improving recognition as well as SA treatment in cardiology might have benefits in the reduction of CV morbidity and mortality [56].

### Limitation and strength of the study

We acknowledge a few *limitations* of this study. First, the cross-sectional design does not permit us to evaluate the longitudinal impact of SA on cardiac structure, as the real duration of the SA is not known. A potential limitation is that our study was performed in a clinic-based sample. Second, in our study we do not separately analyze the influence of central and obstructive events. The causative mechanisms of obstructive and central type sleep apnea differ, but the physiological and hence cardiovascular sequelae can be considered in unison [4, 5, 7].

*The strengths* of this study include relatively large sample size. First, by including a large sample of patients and numerous confounding covariates, our study has overcome many of methodological limitations and provides strong support for an independent cross-sectional association between SA and LV. Second, adjustments of echocardiographic parameters to BMI and hypertension have been successfully implemented. Third, all of our subjects had comprehensive overnight standardized polysomnography data.

## Conclusion

We concluded that among patients with stable CAD, unrecognized SA was highly prevalent, and the majority of these patients did not report excessive daytime sleepiness. Mild to moderate SA was associated with increased LV wall thickness, LV mass, and was associated with higher prevalence of concentric LV geometry independently of coexisting obesity, hypertension, diabetes mellitus or advancing age.

## Abbreviations

ACS: Acute coronary syndromes
AHI: Apnea-hypopnea index
ASE: American Society of Echocardiography
BMI: Body mass index
CV: Cardiovascular
HF: Heart failure
IVST: Interventricular septal thickness
*LV*: Left ventricular or left ventricle
LVEDD: Left ventricular end-diastolic diameter
LVEF: LV ejection fraction
LVESD: LV end-systolic diameter
LVH: LV hypertrophy
LVM: LV mass
LVMI: LV mass index
LVPWT: LV posterior wall thickness
LVWT: LV wall thickness
MI: Myocardial infarction
NYHA: New York Heart Association
ODI: Oxygen desaturation index
OSA: Obstructive sleep apnea
RWT: Relative wall thickness
SA: Sleep apnea
SDB: Sleep disordered breathing.
